# Blepharochalasis: clinical and epidemiological characteristics, surgical strategy and prognosis-- a retrospective cohort study with 93 cases

**DOI:** 10.1186/s12886-021-02049-4

**Published:** 2021-08-28

**Authors:** Jinqiong Zhou, Jingwen Ding, Dongmei Li

**Affiliations:** Beijing Tongren Eye Center, Beijing Tongren Hospital, Beijing Ophthalmology & Visual Science Key Laboratory, Capital Medical University, Beijing, China

**Keywords:** Blepharochalasis, Eyelid diseases, Surgical strategy, Long-term prognosis

## Abstract

**Background:**

Blepharochalasis is a rare eyelid disorder but eventually leading to destructive eyelid deformation. Until now the clinical and epidemiological data are unavailable. This study aimed to report the manifestations, epidemiological characteristics and surgical strategy of a large series of blepharochalasis patients with long-term follow-up. The prognosis of different clinical deformities was also investigated.

**Methods:**

This was a retrospective cohort study, including consecutive patients diagnosed with blepharochalasis in a single center. Blepharoplasty and other surgical approaches were performed according to manifestations, after a 2-year quiescent period with no recurrent attacks and exacerbation of lesions. Prognosis after surgery was recorded.

**Results:**

A total of 93 patients, with a mean age of 30.77 ± 14.04 (range: 9.00–70.00) years were included. Of all those 93 patients, 72.04% were females (67, *P* = 0.02). The mean follow-up was 5.29 ± 2.07 (range: 3–10) years before surgery, and 2.07 (range:1.54–4.22)years follow-up after surgery. The mean age of onset of blepharochalasis symptoms was 10.09 ± 3.32 (range: 5–16) years, and 83.87% patients got symptoms in puberty. With an average of 5 times per year, the mean duration of each acute attack was 28.12 ± 1.01 (rang: 2–192) hours. The mean duration from the onset of acute attack to the quiescent stage lasted for 7.33 ± 2.05 (range: 4–10) years. Most of the cases (88, 94.62%) had more than one manifestation at the end of the last follow-up before surgery. Ptosis (48.39%) was the most common deformity. Followed by lacrimal gland prolapse (44.09%), canthal angle deformity (29.04%), lower eyelid retraction (17.20%). After surgery, the functional and cosmetically acceptable results were achieved in all patients except for overcorrection in 5 (11.90%) patients with ptosis. The lacrimal gland prolapse recurred in two (4.00%) patients at 29 and 36 months after surgery.

**Conclusions:**

Blepharochalasis is rare but mostly occurred in adolescent females. The process from the onset to the stable stage usually lasted for about 7 years, which might be associated with the onset of puberty. Surgical management of clinical manifestations after at least 2-year follow-up period of quiescence would be appropriate in order to observe a great plastic effect, low overcorrection and recurrence rate.

## Background

Blepharochalasis, a rare eyelid disorder that predominantly occurs in young people, is characterized by the onset of eyelid edema and spontaneous resolution, eventually leading to destructive periorbital skin atrophy, deformation and discoloration [1]. Although the disorder has been clinically known for over 200 years, the pathogenesis remains unknown [2]. Till date, there is no large cohort study that dealt with blepharochalasis, and most of the publications were case reports or small series [3–5], as the epidemiological data of blepharochalasis is unavailable. In 2009, Koursh et al. [6] summarized 67 cases of blepharochalasis from 1977 to 2006, describing the natural history, epidemiology, clinical features, etiology, histopathology and pathogenesis, differential diagnosis, treatment and also the prognosis in detailed. However, in this review, the cases were from different races, with short-term follow-up, and the diagnostic criteria and focus remained different. The clinical and epidemiological characteristics, surgical strategy and prognosis of this disorder has not yet been fully elucidated.

In this study, we retrospectively analyzed a large series of consecutive patients with blepharochalasis, and reported the hospital-based epidemiological data, the acute and late clinical manifestations, the surgical strategy and the prognosis of different clinical deformities after surgery.

## Methods

This retrospective cohort study included a group of consecutive patients diagnosed with blepharochalasis from January 2009 to December 2019 in Beijing Tongren Eye Center. All participants or their guardians were fully informed and signed the informed consent, including the agreement for the publication of potentially identifiable information. The study protocol followed the principles of the declaration of Helsinki, and the Medical Ethics Committee of the Beijing Tongren Hospital approved the study.

All participants with blepharochalasis symptoms (including recurrent idiopathic edema and redness in the eyelid or adnexal tissue of the orbit, and other secondary manifestations such as thinned and wrinkled eyelids, ptosis, lacrimal gland prolapse, lids retraction, horizontally shortened palpebral fissure or rounded deformity of the lateral canthal angle) and underwent eyelid plastic surgery were included. Patients were excluded if they had undergone anterior or posterior ocular surgery, had systematic or ocular diseases (such as heredity angioedema or Ascher’s syndrome, thyroid-related ophthalmopathy, floppy eyelid syndrome, orbital cellulitis, sarcoidosis, idiopathic orbital inflammation, hereditary angioedema, dacryoadenitis, lacrimal gland tumor, localized cutis laxa, or any other known ocular diseases), or refused to undergo the eyelid plastic surgery.

All participants underwent an interview by a trained ophthalmologist (JQZ) with standardized questionnaire about the complaint, symptoms, age of the first onset, the characteristics of acute attacks (frequency, duration of each acute attack, the interval between the two attacks), triggering factors, family history, as well as the history of allergies, trauma, and other diseases during their first visit. Age at the onset of puberty was also questioned (the development of secondary sexual characteristics, in females, such as breast and hip growth, and menstruation; In males, testicular development, growth of facial hair, and changes in voice [7]). All participants underwent detailed clinical examinations. The information of the best-corrected visual acuity, eyelid appearance, contour, position, color, deformity of medial or lateral canthal angles, and also the function of levator muscle was recorded [8, 9]. Associated manifestations including ptosis, lacrimal gland prolapse, horizontally shortened palpebral fissure or rounded lateral canthal angle were also recorded.

**Fig. 1:**
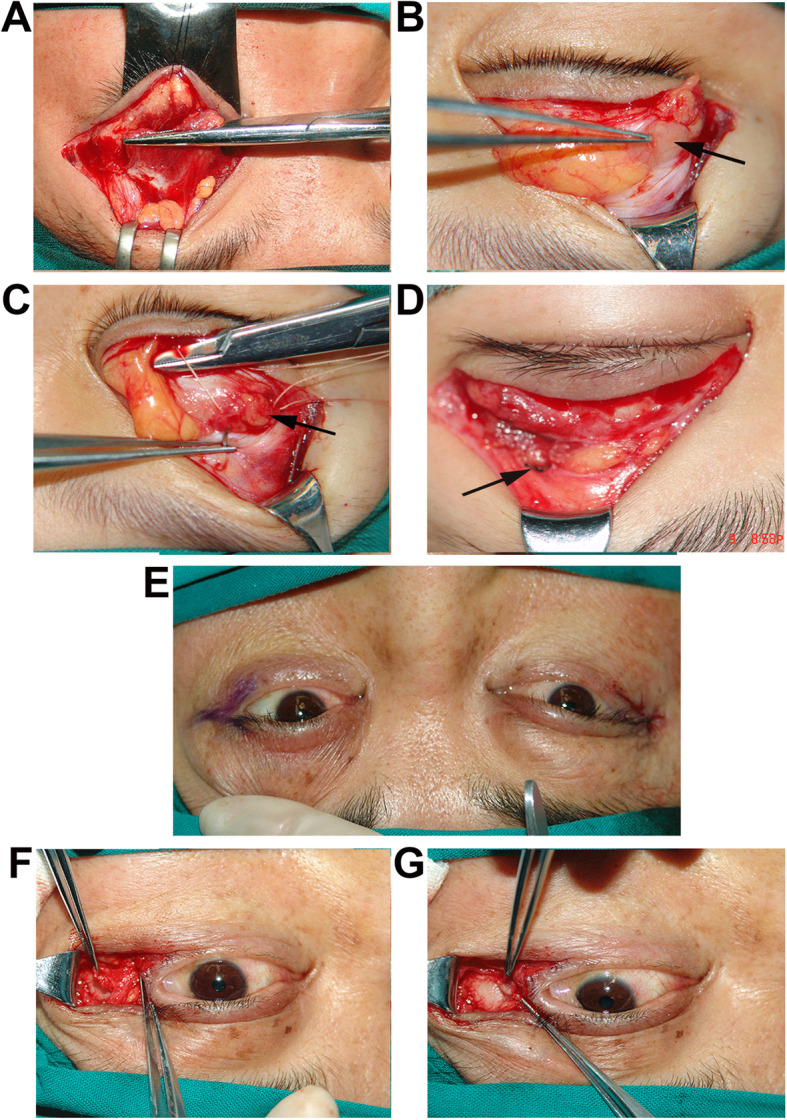
Surgical procedures for the clinical manifestations of blepharochalasis **A**. Showing the levator muscle. **B**. Exposing the prolapsed orbital lacrimal gland (arrow). **C**. Suturing the prolapsed gland (arrow) to the orbital periosteum of the lacrimal fossa. **D**. Suturing the orbital septum (arrow). **E**. Showing the "Y" shaped incision for correcting the lateral canthal deformity. **F**. Showing the isolated periosteal flap. Separating the periosteal flap as upper and lower leaves. **G**. Suturing the periosteal flaps to the appropriate part of the tarsus

After enrollment in our study, all the patients with active blepharochalasis were followed up every 6 months, without any medical treatment. Active blepharochalasis was defined as recurrent episodes of painless and non-erythematous edema of the eyelids in association with telangiectasia [6]. Surgery would be scheduled after at least 2-year quiescent period with no recurrent attack, and followed up for at least 1 year after surgery. All surgical interventions were performed by the same experienced surgeon (DML) under local anesthesia. Surgical approaches varied according to the clinical manifestations of each individual, such as blepharoplasty for wrinkled and redundant eyelids, levator aponeurosis advancement for ptosis, prolapsed lacrimal gland resuspension, canthoplasty for lateral and medial canthal angle deformity. Other surgical approaches were combined according to whether other manifestations were complicated. Eyelid skin excised during blepharoplasty was analyzed histopathologically. The most common surgical procedures of each manifestation were performed as follows:

1. *Blepharoplasty:* all those eyes underwent blepharoplasty, cutting the eyelid skin along the along the lid-crease line, resecting the redundant eyelid lid skin and recreating of the eyelid crease.
2. *Levator aponeurosis advancement*: for eyes with ptosis, cutting off the inner and outer corners of the levator muscle, and also the ligament, which was considered to be important (Figure 1A).
3. *Prolapsed lacrimal gland reposition*: the prolapsed gland was suture repositioned to the orbital periosteum of the lacrimal fossa (Figure 1B, C). The orbital septum was sutured (Figure 1D), and then the redundant lid skin was removed [10].
4. *Canthoplasty*: the lateral canthal deformity was managed by orbital periosteal flap translocation to correct the rounded lateral canthal angle. The “Y” shaped incision was applied (Figure 1E). The tarsus was stretched temporally to the new point to determine the length of the orbital periosteal flap (Figure 1F), and was sutured to the appropriate part of the tarsus (Figure 1G). As for the media canthal deformity, a cross self-drilling tapping titanium nail was used to reattach the media canthal deformity.
5. *Lower eyelid retraction correction:* along the lower eyelid skin incision, Medpore lower lid spacer was used to correct the lower eyelid retraction.
6. *Punctum reposition:* for those eyes combined with punctum outside deformity, surgery for punctum repositioning was performed.

Statistical analysis was performed using a commercially available statistical software package (SPSS for Windows, version 20.0, IBM-SPSS, Chicago, IL). In a first step, we determined the mean values (presented as mean ± standard deviation) and median values of the main outcome parameters. In a second step, we performed student-t test to evaluate the significance of age between unilateral and bilateral patients. The Chi square test was used to assess association between males and females. A *P*-value < 0.05 was considered to indicate statistical significance.

## Results

After excluding 4 patients with ocular diseases (1 with heredity angioedema, 3 with Ascher’s syndrome), and 3 patients without consecutive follow-up after surgery, a total of 93 patients were included in this study. The mean age was 30.77 ± 14.04 (range: 9.00–70.00) years, with 26 males (27.96%) and 67 females (72.04%, *P* = 0.02). The mean age of onset was 10.09 ± 3.32 (range: 5–16) years, and there was no significant difference between genders (*P* = 0.30). Seventy-eight patients (83.87%, 72 females (92%) reported the same year between the onset of blepharochalasis symptoms and the puberty. Twenty-three cases were unilateral (24.73%) and 70 cases (75.27%) were bilateral. The unilaterally affected patients were older than the bilateral ones (11.25 ± 3.59 versus 9.83 ± 3.31 years, *P* = 0.02). However, there was no significant difference between genders (*P* = 0.79). The mean follow-up period was 5.29 ± 2.07(range:3–10) years, with at least 2-year quiescent period before surgery and a mean 2.07-year (range:1.54–4.22) follow-up after surgery.

Six cases (6.45%) reported a known trigger of fever, 2 (2.15%) reported a history of allergies, and others reported no known triggers. None of their family history was contributory. None of the 93 cases reported any other systemic abnormalities. During the early stage, 4 (4.30%) patients were treated with prednisolone (mg/kg/day for 24.21 ± 5.25 days) and 3 (3.23%) patients with acetazolamide (250 mg/day for 15.91 ± 7.54 days) as prescribed by other doctors previously, and the results showed no effective relief from periorbital edema. The vision and ocular movement were normal in all those patients. The results of routine laboratory tests such as blood cell counts, eosinophil count and eythrocyte sedimentation rate were within normal ranges.

With the average of 5 times per year, the frequency of acute attacks was usually exacerbated in the early stage of 2 years, became less frequent as the patients grew up, and eventually entered a relatively quiescent stage. The mean duration of each acute attack was 28.12 ± 1.01 h, ranging from 2 h to 192 h. During the acute attack, the eyelids may present edema without any pressure. Some had complications with conjunctival hyperemia. The frequency of attacks was gradually decreased and disappeared at the late stage. The mean duration from the first onset of acute attack to the quiescent stage lasted for 7.33 ± 2.05 years (range: 4–10 years).

As for clinical manifestations, except for 5 cases presented singly with wrinkled and redundant eyelids, others (88 cases, 94.62%) had more than one manifestation at the end of the last follow-up before surgery. It showed that various clinical manifestations were combined randomly. Wrinkled and redundant eyelids combined with lacrimal gland prolapse was the most common manifestations. Some patients presented more than 5 manifestations.

Of those manifestations, 45 (48.39%) cases presented with ptosis, 41 (44.09%) cases with lacrimal gland prolapse, 20 (21.51%) cases with a rounded deformity of the lateral canthal angle, 7 (7.53%) cases with deformity of the medial canthal angle, 16 (17.20%) cases presented with lower eyelid retraction, 8 (8.60%) cases presented as ectropion accompanied by punctual ectropion. and the remaining 4 cases (4.30%) had retraction in the upper eyelids, presenting as an impression of proptosis. (Table 1).

**Table 1:** Characteristics of the clinical manifestations for eyes with blepharochalasis

	Clinical manifestations							
	wrinkled and redundant eyelids	ptosis	lacrimal gland prolapse	lateral canthal angle deformity	medial canthal angle deformity	lower eyelid retraction	upper eyelid retraction	punctal ectropion
Cumulative cases(n)	93	45	41	20	7	16	4	8
Percent(%)	100	48.39	44.09	21.51	7.53	17.20	4.30	8.60
Males(n)	26	20	5	6	2	6	1	5
Percent(%)	27.96	21.51	5.38	6.45	2.15	6.45	1.08	5.38
*P* value	0.15	0.001	0.002	0.57	0.76	0.18	0.89	0.05

Compared with male patients, ptosis and lacrimal gland prolapse presented more in females, with significant differences (*P* = 0.001 and *P* = 0.002, respectively). As for other manifestations, there was no significant difference between genders (Table 1).

Surgical treatment was performed after at least a 2-year quiescent period with no recurrent attacks and exacerbation of lesions, and at a mean age of 25 ± 3.25 years (range: 16–33 years) old. For those patients, except for 5 patients who underwent only eyelid surgery, all other patients underwent a combination of surgeries. The proportion of patients who underwent each procedure can refer to the distribution of clinical manifestations.

A hematoxylin-eosin stained section of the eyelid skin excised during blepharoplasty showed edema and infiltration of neutrophils in and around the dermal vessels (77/77,100%). Extravasation of erythrocytes was also investigated. Lymphocytes and macrophages were scarcely infiltrated (1/77,1.29%). Elastica van Gieson staining revealed marked decrease of elastic fibers throughout the dermis. The staining of MMP-3 and MMP-9 was observed in and around infiltrating cells in the dermis of some patients (26/77, 33.77%).

**Fig. 2:**
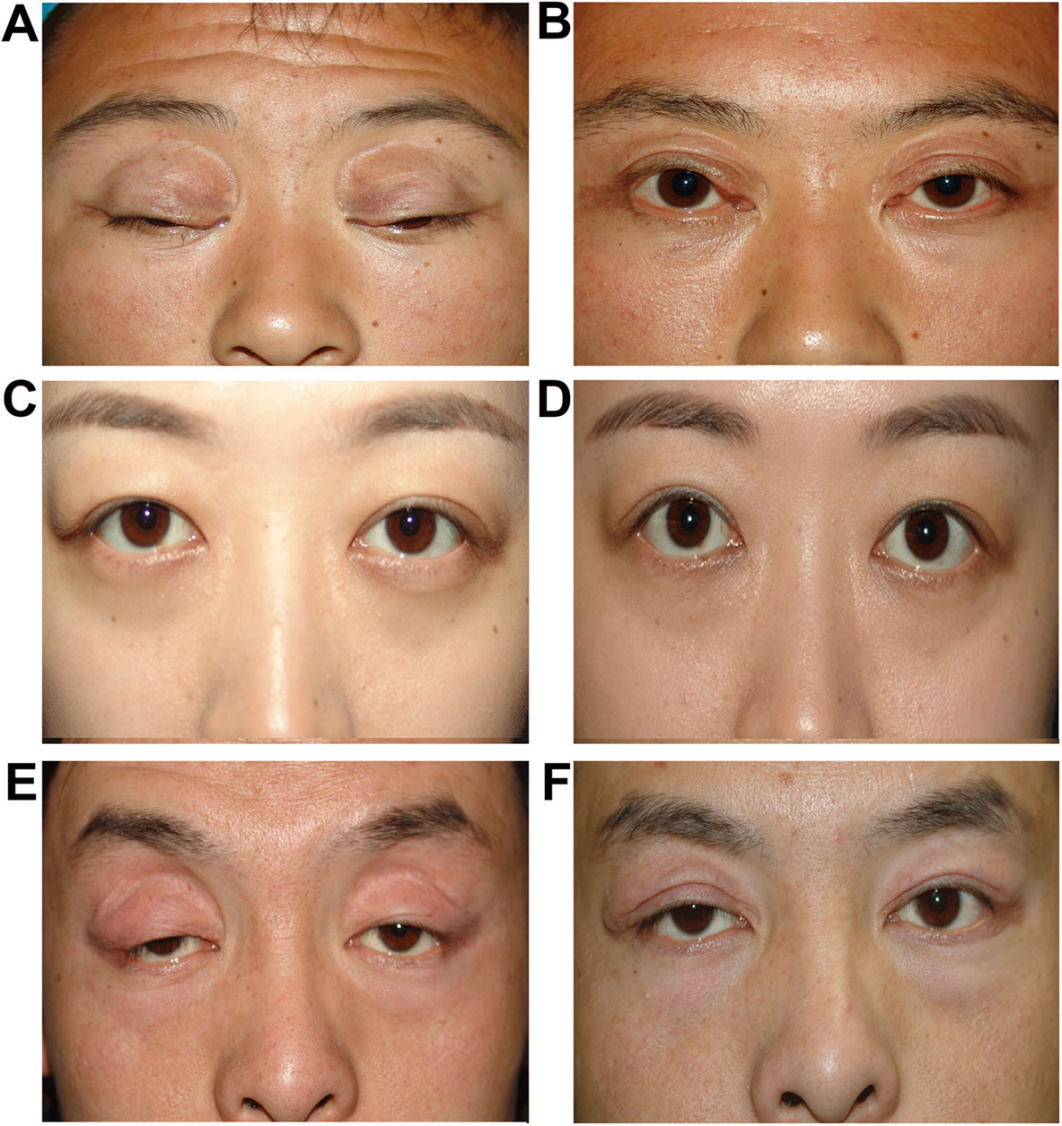
Surgical effects in patients with clinical manifestations of blepharochalasis **A**. Blepharochalasis presented as wrinkled and thinned eyelids affecting both the upper lids, and is associated with ptosis and punctual outside deformity of the upper eyelid. **B**. The same patient of A, one year after surgery of ptosis correction with lid-crease line excision and levator aponeurosis advancement. **C**. Bilateral blepharochalasis is associated with lacrimal gland prolapse. **D**. The same patient of C, 6 months after surgery for prolapsed lacrimal gland reattachment. **E**. Bilateral blepharochalasis associated with ptosis and rounded deformity of the lateral canthal angle. **F**. The same patient of E, one year after surgery for ptosis correction and rounded lateral canthal angle correction

After a 2-year (range: 1–4 years) follow-up postoperatively, all the patients had successful healing of wounds. The position and contour of eyelids and canthus were improved obviously in all patients, with functional and cosmetically acceptable results (Figure 2). Five patients (11.90%) with ptosis presented as overcorrection (written consent for publication were obtained). (Table 2) A second surgery was performed in 4 patients with overcorrection of more than 2 mm to adjust the position of the upper lid in 5 days. The other patient with a mild overcorrection of 1 mm was suggested to undergo a massage of the upper eyelid every day after wound healing. The lacrimal gland prolapse recurred in 2 patients (three eyes) in 29 and 36 months after surgery. Remarkable bulging and mobile firm masses by palpation were revealed in the lateral two-thirds of the upper eyelids. Second surgery was performed to fix the lacrimal gland to the orbital periosteum by silk. No recurrence was noted after following-up for 18 and 24 months, respectively.

**Table 2:** Information about five patients with ptosis presented as overcorrection after surgery

	Age-ranges of onset (years)	Age-ranges of surgery (years)	Degeree of ptosis befor surgery (Cover the cornea mm)	Overcorrection (mm)
Patient 1	14.01–18.00	18.01–30.00	5	2
Patient 2	14.01–18.00	30.01–40.00	3	2
Patient 3	≤12.00	18.01–30.00	6	1
Patient 4	14.01–18.00	18.01–30.00	6	2
Patient 5	14.01–18.00	18.01–30.00	4	4

## Discussion

This hospital-based study showed that blepharochalasis was much less common in males (27.96%) than in females (72.04%). The mean age of onset was 10.09 ± 3.32 years. This study also showed that the symptoms of blepharochalasis occurred bilaterally and symmetrically in most of the patients (75.27%). In this study, the mean process of symptoms would last for about 7 years, with an exacerbation of acute attacks in the early stage of 2 years. The mean age of surgery was 25-years (range: 16–33 years) old. The association between the symptoms and the puberty implied that changes in hormone levels might play an important role in the attack of the disease. Since the available information about blepharochalasis is mainly based on case reports or small case series, the accurate epidemiological data are not available until now. The epidemiological data in this study provided useful information for clinicians to evaluate the clinical course based on specific clinical features and to determine the treatment timing.

As previously reported, in the late stage of the condition, the loss of elastic fibers in the dermis leads to reduced elasticity, which is considered to be a cause of manifestation of blepharochalasis [11, 12]. As the eyelids gradually lose their elasticity, some other manifestations would show up, such as thinned and wrinkled eyelids, ptosis, lacrimal gland prolapse, lids retraction, horizontally shortened palpebral fissure or rounded deformity of the lateral canthal angle. In our study, most of the cases (94.62%) had more than one manifestation at the end of last follow-up. Ptosis (48.39%) was the most common manifestation among these cases. Some scholars hypothesized that the probable site of the lesion was in the aponeurosis or its insertion [13], which required further histopathological support. The second common manifestation was the lacrimal gland prolapse (44.09%), which was occurred due to the atrophic changes of the orbital septum.1 As for the deformity of the lateral or inner canthal angle, the dehiscence of attachments of upper and lower eyelids to the lateral position and occasionally medial canthal tendons were considered as the main causative reasons [14].

There was no evidence-based data for the guidance of treatment of blepharochalasis [2, 15]. Surgical management for clinical manifestations remains to be the primary choice. Surgery should be performed when the disease was in a quiescent phase to avoid recurrent bouts or new episodes. Except for those certain cases with functional vision problems or severe cosmetic disturbances, the patients need early treatment, and surgical treatment is performed in those patients with symptoms quiet for 6--12 months before undergoing the treatment. However, based on our clinical experience, the surgical treatment should be scheduled after at least 2-year quiescent period, as it could reduce the recurrence and achieve the best prognosis. Our previous study proved it once again.

Even though there was limited available data in relevant reports for us to compare, a published review had summarized the postoperative results [5]. The review stated that surgery in blepharochalasis patients was complicated by a high incidence of overcorrection. There were 5 cases that presented overcorrection in this study, much lower than that in previous reports. First of all, ptosis in blepharochalasis is secondary to dehiscence of the levator aponeurosis, whereas the levator function itself is conserved, which is different from congenital ptosis. If the surgical protocol of congenital ptosis was followed, then overcorrection would be inevitable. Secondly, Since recurrent bouts of edema and stretching resulting in inappropriate adhesion and innervation of the lower portion of aponeurosis, higher overcorrection might be secondary to the difficulty in identifying and disconnecting the aponeurotic disinsertion. Thirdly, we assumed that surgery under local anesthesia was much better than under general anesthesia. This is because the function of levator muscle in most of the patients could be assessed much better during the surgery than preoperative assessment according to our experience. With the cooperation of patients, the surgeon can directly observe the function of levator muscle intraoperatively in a real-time manner, which might be much more precise to assess the effect of surgery than estimating under general anesthesia. All of our surgical interventions were performed under local anesthesia.

As the eyelids gradually lose their elasticity, and develop orbital fat atrophy later, the recurrence was common in patients with prolapsed lacrimal gland after surgery. Even though the result was much better than other studies [6], there were two patients (3 eyes) who had recurrence in this study. We supposed that paying attention to fix the gland to the orbital periosteum and suture the orbital septum during the surgery remained crucial to avoid recurrence. In addition, recurrence was also the main problem for patients with lateral canthal deformity after undergoing surgery with canthal ligament suspension, and this may be because of the canthal ligament degeneration [16]. In this study, we applied the orbital periosteal flap to fix the lateral canthal, achieving much better appearance.

However, potential limitations of our study should be considered. Firstly, this is a retrospective study conducted in a single-center, without double-blindness, and selection bias could have accentuated some estimates and masked others. Secondly, because of the population-based study, referral bias cannot be ruled out. Thirdly, there are few indicators in this observation, and lacked functional prognostic indicators. Fourthly, the follow-up time is relatively short, and long-term follow-up on the prognosis is warranted. Fifthly, All those patients in our study belong to the same ethnic (Han) in China. So we didn’t analyze the difference in clinical manifestations between the ethnic groups.

## Conclusion

In conclusion, blepharochalasis occurred mainly in adolescent females, with a mean onset age of 10 years. The process from the onset to the stable stage usually lasted for about 7 years, and is closely associated with puberty. Surgical management of clinical manifestations after at least 2-year quiescent period was recommended. Further histopathological examinations in early and late stages of the disease should be performed to better assess the pathogenesis of the disease.

## Data Availability

The datasets used and/or analysed during the current study are available from the corresponding author on reasonable request.
